# GroEL triggers NLRP3 inflammasome activation through the TLR/NF-κB p-p65 axis in human periodontal ligament stem cells: GroEL triggers NLRP3 inflammasome activation

**DOI:** 10.3724/abbs.2024050

**Published:** 2024-04-10

**Authors:** Li Zhang, Mengmeng Duan, Xiaohua Pu, Huiling Zheng, Xinjie Ning, Ying Tu, Chunming Xu, Demao Zhang, Chengcheng Liu, Jing Xie

**Affiliations:** 1 State Key Laboratory of Oral Diseases & National Center for Stomatology & National Clinical Research Center for Oral Diseases West China Hospital of Stomatology Sichuan University Chengdu 610041 China; 2 School of Basic Medicine Gannan Medical University Ganzhou 341000 China

**Keywords:** GroEL, NLRP3 inflammasome, TLR, NF-κB signaling, periodontitis

## Abstract

The interaction between bacteria and the host plays a vital role in the initiation and progression of systemic diseases, including gastrointestinal and oral diseases, due to the secretion of various virulence factors from these pathogens. GroEL, a potent virulence factor secreted by multiple oral pathogenic bacteria, is implicated in the damage of gingival epithelium, periodontal ligament, alveolar bone and other peripheral tissues. However, the underlying biomechanism is still largely unknown. In the present study, we verify that GroEL can trigger the activation of NLRP3 inflammasome and its downstream effector molecules, IL-1β and IL-18, in human periodontal ligament stem cells (hPDLSCs) and resultantly induce high activation of gelatinases (MMP-2 and MMP-9) to promote the degradation of extracellular matrix (ECM). GroEL-mediated activation of the NLRP3 inflammasome requires the participation of Toll-like receptors (TLR2 and TLR4). High upregulation of TLR2 and TLR4 induces the enhancement of NF-κB (p-p65) signaling and promotes its nuclear accumulation, thus activating the NLRP3 inflammasome. These results are verified in a rat model with direct injection of GroEL. Collectively, this study provides insight into the role of virulence factors in bacteria-induced host immune response and may also provide a new clue for the prevention of periodontitis.

## Introduction

Periodontitis, a microbiome-driven chronic inflammatory disease, occurs in periodontal tissue and can lead to persistent destruction of periodontal tissue and eventually tooth loss [ 1, 2] . Severe periodontitis is the sixth most prevalent disease worldwide and affects approximately 10% of adults globally [3]. In addition, periodontitis is closely related to the occurrence and development of systemic diseases, such as diabetes, cardiovascular disease, Alzheimer’s disease and rheumatoid joints [ 1, 4, 5] . Pathogenic bacteria in subgingival plaques are a precondition for periodontitis [ 6, 7] . Pathogenic bacteria and their microbial virulence factors, such as lipopolysaccharide (LPS), proteases, GroEL and lipoteichoic acid, stimulate host cells to produce inflammatory factors and enzymes, which in turn contribute to the destruction of periodontal tissues [8].

Bacterial GroEL, a member of the heat shock protein (HSP) family, is highly homologous to human HSP60 and plays an important role in the proper folding and translocation of proteins during protein synthesis, as well as in the recombination of denatured proteins [ 9, 10] . Nonetheless, GroEL has also been reported to play a pathogenic role in a variety of bacterial infectious diseases and autoimmune diseases [ 11‒ 13] . Our previous study revealed that GroEL levels in gingival crevicular fluid and saliva were higher in patients with apical periodontitis than in healthy individuals [14]. A previous study based on clinical investigation reported that salivary immunoglobulin A (IgA) has immunoreactivities with *Porphyromonas gingivalis* ( *P*. *gingivalis*) GroEL and *Campylobacter rectus* ( *C*. *rectus*) GroEL, and its level is correlated with the severity of periodontal disease [15]. Another study reported that elevated levels of anti-HSP60 antibodies were detected in the serum of cardiovascular patients with severe periodontitis [16]. Meanwhile, periodontal therapy could significantly reduce the GroEL expression level in serum derived from *P*. *gingivalis* [17]. Local injection of bacterial GroEL induces absorption of the skull in mice and alveolar bone in rats [ 11, 18] . In addition, GroEL from *Tannerella forsythia (T*. *forsythia)* and *C*. *rectus* promotes the production of interleukin 6 and 8 in human gingival fibroblasts and periodontal ligament fibroblasts (hPDLFs) [ 11, 19] . Lin *et al*. [20] reported that *P*. *gingivalis* GroEL induced the production of IL-6 and IL-8 in osteoblasts through p38/MAPK or JNK/MAPK signaling. *Fusobacterium nucleatum* ( *F*. *nucleatum*) GroEL significantly upregulated the expressions of adhesion molecules such as intercellular adhesion molecule-1 (ICAM-1), vascular cell adhesion molecule-1 (VCAM-1) and E-selectin, as well as chemokines such as IL-8 and monocyte chemoattractant protein-1 (MCP-1), in human microvascular endothelial cells and enhanced the adhesion and migration of monocytes [21]. Previous evidence strongly suggested that GroEL may be a potential stimulator of periodontal disease. However, its virulent effect on periodontitis is not fully understood.

Inflammasomes, sophisticated supramolecular complexes in the cytoplasm, are essential for the innate immune responses against pathogens and endogenous cellular damage. One of the most representative inflammasomes is the Nod-like receptor family pyrin domain-containing protein 3 (NLRP3) inflammasome, which consists of NLRP3, apoptosis-associated speck-like protein containing a caspase-1 recruitment domain (ASC), and pro-caspase-1 (the effector) [22]. An increasing number of studies have indicated a role for the NLRP3 inflammasome in the periodontal immune response [ 23‒ 25] . A clinical report showed that periodontitis patients have higher levels of NLRP3, ASC, caspase 1 and IL-1β in serum and saliva than healthy controls [23]. In a rat root resorption (RR) model, increases in M1 macrophages, NLRP3, caspase 1 and IL-1β were detected in the periodontal ligament near the RR regions, and force-treated hPDLCs stimulated M1 macrophage polarization and IL-1β production through NLRP3 inflammasome activation [25]. In a ligature-induced periodontitis model, compared with wild-type mice, *NLRP3*-knockout mice exhibited decreased loss of alveolar bone, reduced release of IL-1β and impaired differentiation of osteoclasts [26]. In addition, several studies have reported that the NLRP3 inflammasome is activated by pathogenic stimuli that act on periodontal tissue cells, including hPDLFs and macrophages [ 27, 28] . Lu *et al*. [28] reported that muramyl dipeptide (MDP) and LPS from *Escherichia coli* ( *E*. *coli*) induced NLRP3 inflammasome activation and IL-1β secretion in an ASC-dependent manner in hPDLFs.

Although GroEL is an important virulence factor implicated in bacteria-host interactions, the effect of GroEL on NLRP3 inflammasome in periodontal tissue is still unclear. Thus, in this study, we investigated the role of GroEL in NLRP3 inflammasome both in hPDLSCs *in vitro* and in rat periodontal tissues *in vivo*, and elucidated the underlying mechanisms involved.

## Materials and Methods

### Cell culture and characterization

hPDLSCs were obtained as reported in a previous study [29]. The procedures were approved by the Human Research Ethics Committee of West China Hospital of Stomatology, Sichuan University (WCHSIRB-D-2020-048). In brief, we collected 16 human caries- and periodontal disease-free adult premolars extracted for orthodontic treatment (aged 12–24 years). We then obtained the periodontal ligaments of the middle third of the roots. Next, the periodontal ligament samples were incubated with 3 mg/mL collagenase I (17100017; Gibco, Carlsbad, USA.) solution at 37°C. After incubation for 30 min, the collagenase I solution was removed by centrifugation (1000 rpm, 5 min). Subsequently, the periodontal ligament samples were cultured in a culture flask with α-MEM containing 10% fetal bovine serum (FBS) and 1% penicillin-streptomycin in an incubator with 5% CO _2_ at 37°C. When the cells reached approximately 80% confluence, a subculture was performed, and hPDLSCs at passages 3–5 were used in subsequent experiments. The stemness of isolated hPDLSCs was confirmed by alizarin red, alkaline phosphatase and Oil Red O staining using standard protocols.

### CCK8 assay

The cytotoxicity of GroEL, ST2825, and BAY11-7082 on hPDLSCs was explored first to determine their appropriate concentrations for the subsequent experiments. hPDLSCs were seeded in 96-well plates and treated with GroEL (c7688; Sigma-Aldrich, St Louis, USA) at 0.5, 1, 5, 10 and 25 μg/mL for 48 h, or with ST2825 (HY-50937; MCE, Monmouth Junction, USA) at 0, 1, 3, 5, 10 and 20 μM for 24 h, or BAY11-7082 (HY-13453; MCE) at 0, 1, 3, 5, 10 and 20 μM for 24 h. The viability of cells was determined using a CCK8 assay kit (CK04-500T; Dongren, Shanghai, China) according to the manufacturer’s protocol.

### GroEL treatment

hPDLSCs were seeded in 6-well plates at 2.5×10 ^5^ cells/well and cultured with 10% FBS in α-MEM till cells were adherent. The medium was changed to α-MEM containing 2% FBS for starvation for 12 h. Then cells were stimulated with different concentrations (0, 1 or 10 μg/mL) of GroEL. For RT-PCR analysis, cells were collected after 2 or 12 h of GroEL treatment. For western blot analysis, cell lysate samples were harvested after 3, 6, 12 or 24 h of GroEL treatment. Cell culture supernatant samples were collected at 12, 24, 48 and 72 h for zymography analysis. For immunofluorescence analysis, hPDLSCs were seeded onto glass bottom dishes (5×10 ^3^ cells/well) and cultured in α-MEM supplemented with 10% FBS. Then, cells were stimulated with 10 μg/mL GroEL for 6 or 24 h with or without pretreatment with 5 μM of ST2825 or 5 μM BAY11-7082 for 1 h.

### Quantitative real-time polymerase chain reaction (qPCR)

Total RNA was harvested from hPDLSCs using an RNA fast isolation kit (RP1202; BioTeke, Beijing, China) following the manufacturer’s guidelines, and then reverse transcribed into cDNA using Hifair III 1st Strand cDNA Synthesis SuperMix (11137ES60; YEASEN, Shanghai, China). Next, cDNA, forward primer, reverse primer, nuclease-free water and qPCR SYBR Green Master Mix (11198; YEASEN) were mixed to form a reaction mixture, and qRT-PCR was subsequently carried out on a CFX Connect Real-Time System (Bio-Rad, Hercules, USA). The detailed program settings were made according to the manufacturer’s instructions. Using *GAPDH* as an internal control, the 2 ^‒ΔΔCt^ method was utilized to analyze the expressions of target genes. The primer sequences for the genes in this investigation are listed in Supplementary Table S1.

### Western blot analysis

Total protein was extracted from hPDLSCs using a radioimmunoprecipitation assay buffer (RIPA; 68117726; Biosharp, Guangzhou, China) containing 1% protease inhibitor. The protein concentration of the samples was determined using BAC assay kit (P0010; Beyotime, Shanghai, China). Following denaturation, the proteins were separated by SDS-polyacrylamide gel electrophoresis (SDS-PAGE) and then transferred to PVDF membranes. After being blocked, the membranes were incubated with specific primary antibodies (1:1000) against the following proteins: NLRP3 (AG-20B-0014-C100; AdipoGen, San Diego, USA), caspase 1 (HY-P80622; MCE), β-actin (T200068-8F10; Zen-Bio, Chengdu, China), IL-1β (RB20040UC; Zen-Bio), IL-18 (10663-1-AP; Proteintech, Chicago, USA), MMP-2 (ab97779; Abcam, Cambridge, UK), IL-6 (500286; Zen-Bio), MMP-9 (ab38898; Abcam), TNFα (346654; Zen-Bio), NF-κB p65 (250060; Zen-Bio), phospho-NF-κB p65 (3033; Cell Signaling Technology, Beverly, USA), p38 (340697; Zen-Bio), phospho-p38-MAPK (310091; Zen-Bio), JNK (380556; Zen-Bio), phospho-JNK1 (381100; Zen-Bio), ERK1+ERK2 (ab184699; Abcam), and phospho-ERK1+phospho-ERK2 (ab201015; Abcam). Next, the membranes were treated with secondary antibody for 2 h. Protein bands were visualized using a ChemiDoc ^TM^ Touch Imaging System (Bio-Rad). ImageJ was used to analyze the band intensity.

### Gelatin zymography

Zymography was performed using the protocol described in a previous study [14]. BAC assay was used to detect the protein concentration of the cell culture supernatant samples, which were then mixed with loading buffer. Then, equal amounts of samples were loaded onto gels containing gelatin SDS to separate the proteins. Next, the gels were washed with 2.5% Triton X-100 solution three times and incubated with proteolysis buffer overnight at 37°C. Coomassie blue solution was applied to stain the gels for approximately 2 h, after which destaining buffer was added. The protein bands were visualized using a ChemiDocTM Touch Imaging System (Bio-Rad) and the band intensity was quantified with ImageJ software.

### Animal experiment

Ten male SD rats (6 weeks old) were purchased from the Animal Experiment Center of Sichuan University. The experimental design was approved by the Institutional Animal Care and Use Committee (WCHSIRB-D-2021-601). The rats were randomly assigned to the control group or GroEL group ( *n*=5). Rats in the GroEL treatment group were administered with 10 μL of GroEL (1 μg/μL) into the distal and mesial interdental gingiva of the maxillary second molar, respectively. Rats in the control group were administered with PBS into both the distal and mesial interdental gingiva of the maxillary second molar. The injections were performed 3 times per week. Four weeks after injection, all rats were euthanized by cervical dislocation under isoflurane inhalation anesthesia, and the maxilla was collected and analyzed. The statistical analyses of different experiments were based on the actual number of samples used.

### Microcomputed tomography (μ-CT)

All maxillary specimens were scanned with a μ-CT scanner (Scanco, Wangen-Bruttisellen, Switzerland) operating at 200 μA and 70 kV, with an exposure time of 300 ms and a resolution of 10 μm. 3D reconstructions of the maxillary region were generated with the SCANCO Medical evaluation program. The distance from the cementoenamel junction (CEJ) to the alveolar bone crest (ABC) of the maxillary second molar was measured and is expressed as the CEJ-ABC.

### Hematoxylin and eosin (H&E) staining

After dewaxing, maxillary sections (5 μm) were hydrated using graduated alcohol baths and double-distilled water. Tissue sections were stained with hematoxylin and then successively immersed in HCl-EOTH and ammonia-H _2_O to separate colors. Next, the sections were stained with hematoxylin and eosin. Images were captured using a microscope (BX53; Olympus, Tokyo, Japan).

### Masson staining

After dewaxing in water, maxillary sections (5 μm) were stained with Masson staining solution (G1340; Solarbio, Beijing, China) following the manufacturer’s instructions. Then, the sections were dehydrated and sealed with resin. Images were captured using a microscope (BX53; Olympus).

### Immunofluorescence analysis

Immunofluorescence staining was performed as described previously [ 30, 31] . Briefly, hPDLSCs were rinsed with PBS, fixed with 4% cold paraformaldehyde (PFA), permeabilized with 0.5% Triton X-100 solution and then blocked with 5% BSA for 1 h. Then, cells were incubated with primary antibodies (1:200) against NLRP3 (AG-20B-0014-C100; AdipoGen) and phospho-NF-κB p65 (3033; Cell Signaling Technology) overnight at 4°C. The samples were treated with fluorescein-conjugated anti-rabbit secondary antibody (1:200; ab150077; AbcamK) for 2 h after being washed with PBS. The cytoskeleton and nuclei were detected by counterstaining with FITC-phalloidin (Invitrogen, Carlsbad, USA) and DAPI (D9542; Sigma-Aldrich), respectively. Fluorescence images were obtained with a confocal laser scanning microscope (CLSM, FV3000; Olympus). The fluorescence intensities of NLRP3 and NF-κB (p-p65) were analyzed by ImageJ software.

After dewaxing in water, the maxillary sections underwent heat-induced epitope retrieval. Next, the sections were exposed to 3% H _2_O _2_ for 30 min and blocked with 5% BSA solution for 1 h. Then, the sections were incubated overnight with primary antibodies (1:200): anti-NLRP3, anti-IL-1β, anti-IL-18, anti-MMP-2, anti-IL-6, anti-MMP-9, anti-TNFα and anti-phospho-NF-κB p65. The sections were treated with fluorescein-conjugated anti-rabbit IgG secondary antibody for 2 h at room temperature. DAPI was used to visualize the nuclei. Fluorescence images were obtained with a microscope slide scanner (SLIDEVIEW VS200; Olympus).

### Statistical analysis

The data in this study are expressed as the mean±SD. One-way analysis of variance (ANOVA) with a Bonferroni post hoc correction and Student’s *t* test were performed using GraphPad Prism version 9 software to assess group differences. *P*<0.05 was considered statistically significant.

## Results

### GroEL activates the NLRP3 inflammasome

To ensure the stemness of isolated hPDLSCs, we first confirmed that hPDLSCs had multilineage differentiation capability by using alizarin red, alkaline phosphatase and Oil Red O staining ( Supplementary Figure S1A‒C). Meanwhile, hPDLSCs stably expressed CD90 and Cd146 ( Supplementary Figure S1D). To explore the cytotoxicity of GroEL on hPDLSCs, CCK8 assay was performed, and it was found that the viability of hPDLSCs was not significantly affected by GroEL at different concentrations (0.5, 1, 5, 10 or 25 μg/mL) within 48 h ( Supplementary Figure S2).

**Figure 1:**
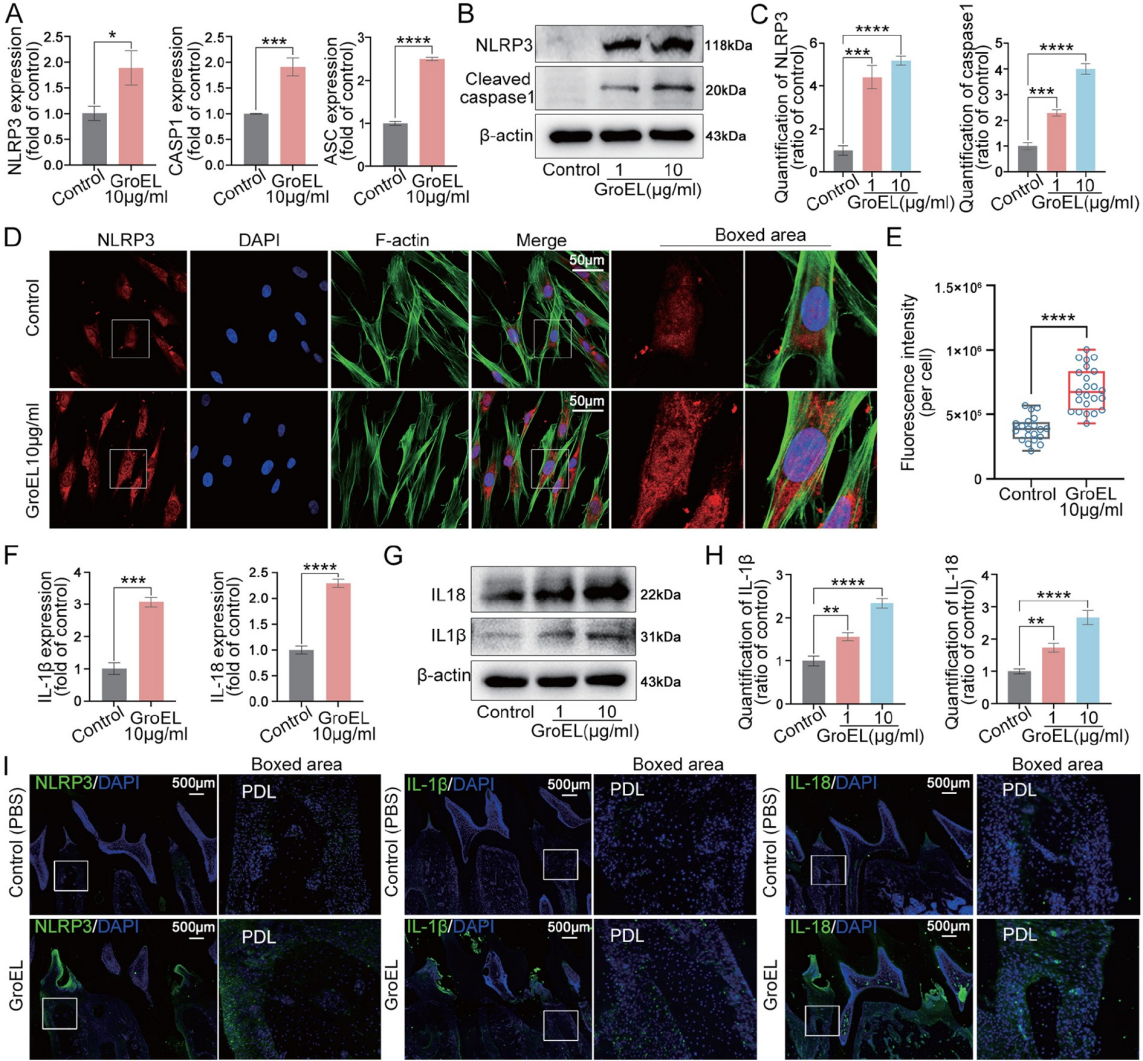
Figure 1 GroEL activates the NLRP3 inflammasome in hPDLSCs *in vitro* and *in vivo* (A) qPCR showing the mRNA expressions of NLRP3, ASC and CASP1 in hPDLSCs stimulated with GroEL. The data were derived from three separate experiments ( n=3). * P<0.05, *** P<0.001, **** P<0.0001. (B) Western blot images showing the protein expressions of NLRP3 and cleaved caspase-1 in hPDLSCs treated with GroEL. The images were derived from three separate experiments ( n=3). (C) Quantitative analysis confirming the changes in the levels of the NLRP3 and cleaved caspase-1 proteins in (B). Relative protein expression was normalized to that of β-actin. The data were derived from three separate experiments ( n=3). *** P<0.001, **** P<0.0001. (D) Immunofluorescence images showing the increased expression of NLRP3 in hPDLSCs treated with 10 μg/mL GroEL. The data were derived from three separate experiments (n=3). NLRP3, red; F-actin, green; nuclei, blue. (E) Quantitative analysis showing the increase in the protein level of NLRP3 in (D) according to the total fluorescence density (OD/cell area). The analysis was based on 20 cells from three separate experiments ( n=3). **** P<0.0001. (F) qPCR showing the mRNA expressions of IL-1β and IL-18 in hPDLSCs stimulated with GroEL. The data were derived from three separate experiments ( n=3). *** P<0.001, **** P<0.0001. (G) Western blot images showing the protein expressions of IL-1β and IL-18 in hPDLSCs treated with GroEL. The data were chosen from three separate experiments ( n=3). (H) Quantitative analysis confirming the changes in the levels of the IL-1β and IL-18 proteins in (G). Relative protein expression was normalized to that of β-actin. The data were derived from three separate experiments ( n=3). ** P<0.01, **** P<0.0001. (I) Immunohistofluorescence images showing the increased expressions of NLRP3, IL-1β and IL-18 in rat periodontal tissues 4 weeks after the injection of GroEL. NLRP3, IL-1β and IL-18, green; nuclei, blue. The data were derived from four separate experiments ( n=4).

Then, we investigated the influence of GroEL, an important virulence factor implicated in bacteria-host interaction, on the initiation of inflammation in hPDLSCs. We found that GroEL could upregulate the gene expressions of NLRP3, CASP1 and ASC, which are the three main components of the NLRP3 inflammasome [22], in hPDLSCs after treatment with 10 μg/mL of GroEL for 12 h ( Figure 1A). We then detected the expressions of these proteins by western blot analysis, and the results demonstrated that GroEL increased the protein expressions of NLRP3 and cleaved caspase 1 in hPDLSCs ( Figure 1B). Quantitative analysis confirmed the increases in the protein levels of NLRP3 and cleaved caspase 1 ( Figure 1C). We next investigated the cytoplasmic expression and distribution of NLRP3 by immunofluorescence microscopy, and the results showed that NLRP3 expression was largely enhanced in hPDLSCs after treatment with 10 μg/mL of GroEL for 24 h ( Figure 1D). Quantitative analysis based on total fluorescence intensity (per cell) confirmed the changes in the protein expression of NLRP3 ( Figure 1E). The activation of the NLRP3 inflammasome could promote the release of cleaved caspase 1 (the active form of caspase 1) to trigger the expressions of the downstream effector molecules IL-1β and IL-18 [25]. Thus, we detected changes in the expressions of IL-1β and IL-18 in hPDLSCs induced by GroEL. qPCR results revealed that treatment with 10 μg/mL of GroEL upregulated the expressions of IL-1β and IL-18 ( Figure 1F). At the protein level, we found that GroEL significantly increased the expressions of IL-1β and IL-18, as determined by western blot analysis ( Figure 1G,H). We directly injected GroEL (10 μg per rat, 3 times per week) into rat periodontal tissues for 4 weeks and investigated the impact of GroEL on the periodontium at the tissue level ( Figure 1I). The results showed that higher expression levels of NLRP3, IL-1β and IL-18 were detected in the GroEL-treated group compared to those in the control group.

In addition, treatment with GroEL also increased the expressions of inflammatory factors IL-6 and TNFα in hPDLSCs ( Supplementary Figure S3). At the mRNA level, the expressions of IL-6 and TNFα were upregulated in hPDLSCs after treatment with 10 μg/mL of GroEL for 12 h ( Supplementary Figure S3A). At the protein level, increased expressions of IL-6 and TNFα were detected in hPDLSCs after treatment with 10 μg/mL of GroEL for 24 h ( Supplementary Figure S3B,C). At the tissue level, increased expressions of IL-6 and TNFα were also detected in rat periodontal tissues after treatment with GroEL for 4 weeks ( Supplementary Figure S3D).

### GroEL increases the activities of MMP-2 and MMP-9

**Figure 2:**
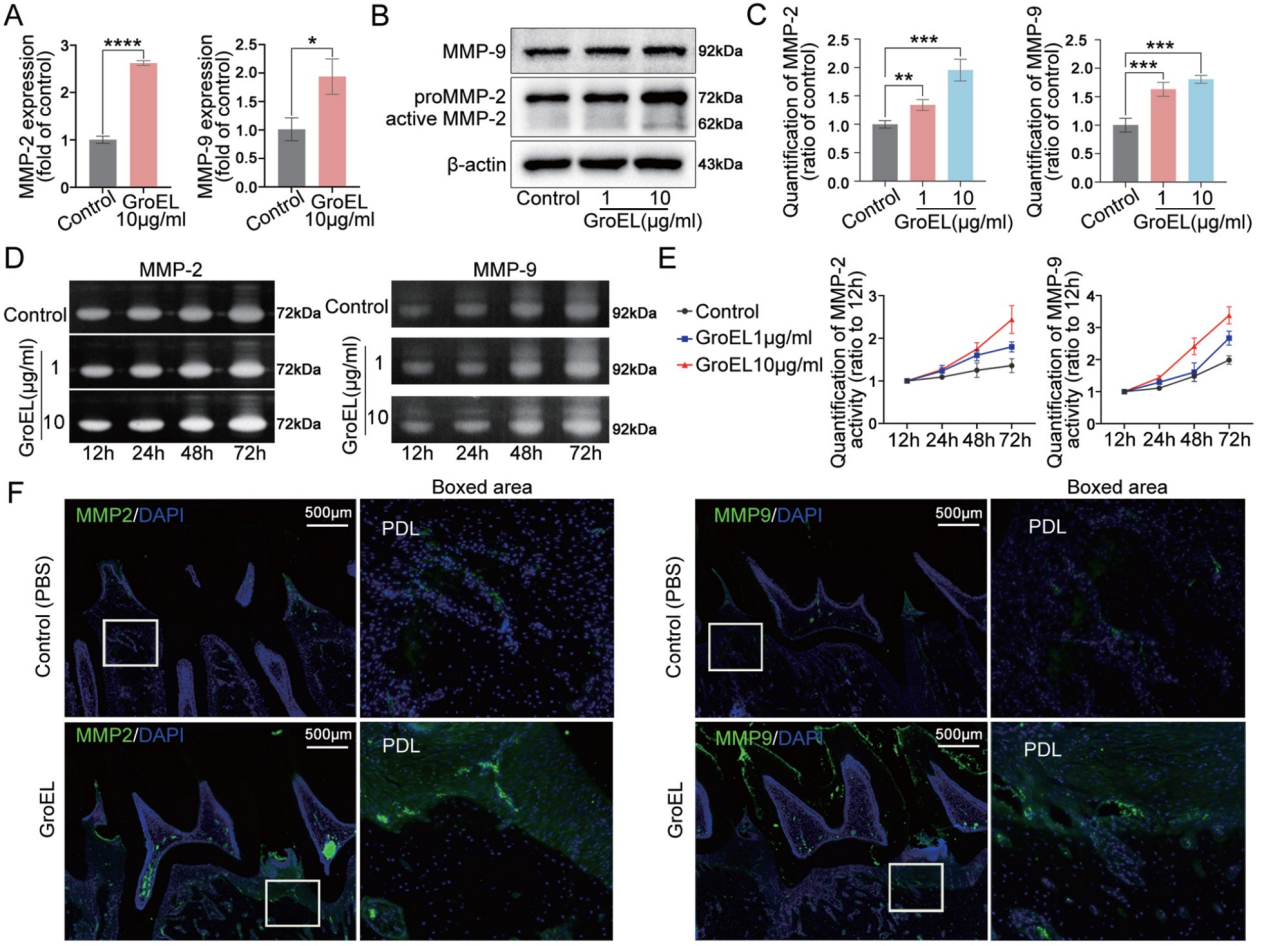
Figure 2 GroEL increases MMP-2 and MMP-9 activities in hPDLSCs both *in vitro* and *in vivo* (A) qPCR showing the mRNA expressions of MMP-2 and MMP-9 in hPDLSCs induced by GroEL. The data were derived from three separate experiments ( n=3). * P<0.05, **** P<0.0001. (B) Western blot images showing the protein expressions of MMP-2 and MMP-9 in hPDLSCs treated with GroEL. The images were chosen from three separate experiments ( n=3). (C) Quantitative analysis confirming the changes in the levels of the MMP-2 and MMP-9 proteins in (B). Relative protein expression was normalized to that of β-actin. The data were derived from three separate experiments ( n=3). ** P<0.01, *** P<0.001. (D) Gelatin zymography images showing the increased activities of MMP-2 and MMP-9 in GroEL-treated hPDLSCs. The images are based on three separate experiments ( n=3). (E) Quantitative analysis confirming the increased activities of MMP-2 and MMP-9 in (D). The data were derived from three separate experiments ( n=3). (F) Immunohistofluorescence images illustrating the increased expression of MMP-2 and MMP-9 in rat periodontal tissues 4 weeks after the injection of GroEL. MMP-2 and MMP-9, green; nuclei, blue. The data were derived from four separate experiments ( n=4).

The gelatinases MMP-2 and MMP-9 are extracellular matrix (ECM)-degrading enzymes that play indispensable roles in connective tissue remodeling [32]. By using qPCR analysis, we found that GroEL could upregulate the gene expressions of *MMP-2* and *MMP-9* in hPDLSCs ( Figure 2A). At the protein level, the expression levels of MMP-2 and MMP-9 were increased in hPDLSCs after treatment with GroEL, as detected by western blot analysis ( Figure 2B). Quantitative analysis confirmed the increases in the MMP-2 and MMP-9 proteins ( Figure 2C). To verify the activities of secreted MMP-2 and MMP-9, we collected culture media and performed gelatin zymography assay, and the results showed that GroEL increased the activities of MMP-2 and MMP-9 in hPDLSCs at concentrations of 1 and 10 μg/mL ( Figure 2D). Quantification results revealed time-dependent increases of MMP-2 and MMP-9 in hPDLSCs induced by GroEL within 72 h ( Figure 2E). Moreover, the expression levels of MMP-2 and MMP-9 were increased by up to 1.8-fold and 1.7-fold, respectively, in hPDLSCs treated with GroEL (10 μg/mL) for 72 h ( Supplementary Figure S4). At the tissue level, we detected the expressions of MMP-2 and MMP-9 by immunofluorescence microscopy, and the results indicated that GroEL induced higher expressions of MMP-2 and MMP-9 in the rat periodontium compared to those in the non-treated control group rats ( Figure 2F). Additionally, we detected the expressions of tissue inhibitors of metalloproteinases (TIMPs) which are endogenous inhibitors of MMPs [33], and found that the mRNA expression levels of *TIMP-1* and T *IMP-2* were significantly decreased in hPDLSCs treated with GroEL (10 μg/mL) for 12 h ( Supplementary Figure S5). Taken together, these results indicate that GroEL has a great impact on ECM degradation by upregulation of MMP-2 and MMP-9 expressions.

### GroEL activates NF-κB signaling in hPDLSCs

**Figure 3:**
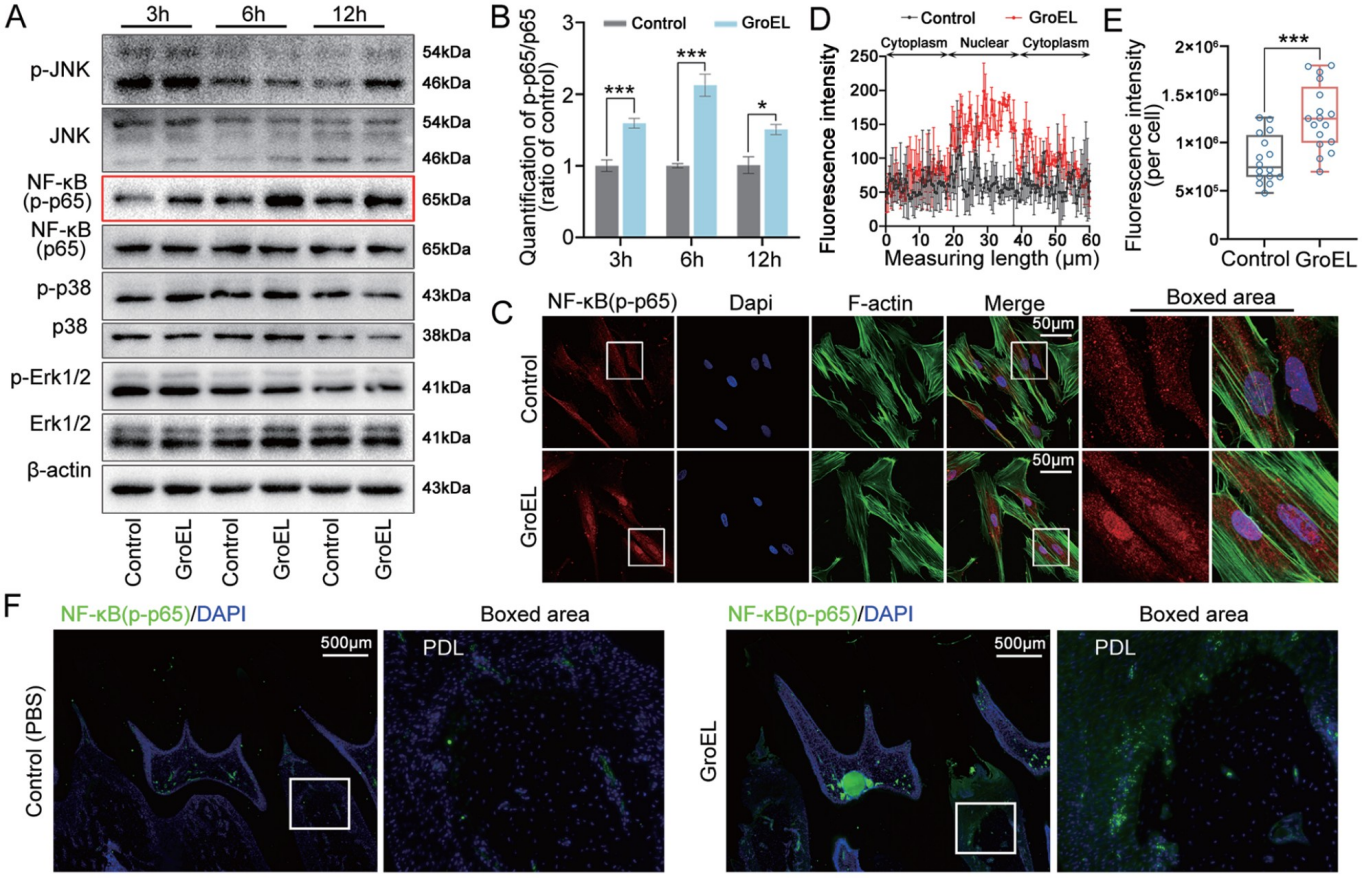
Figure 3 GroEL activates NF-κB signalling both *in vitro* and *in vivo* (A) Western blot analysis showing the protein expressions of JNK, NF-κB, p38 and Erk1/2 in hPDLSCs induced by GroEL. hPDLSCs were treated with 10 μg/mL GroEL for 3, 6, or 12 h. Images were obtained from three separate experiments ( n=3). (B) Quantitative analysis confirming the increased expression of NF-κB (p-p65/p65) in (A). Relative protein expression was normalized to that of β-actin. The data were analyzed based on three separate experiments ( n=3). * P<0.05, *** P< 0.001. (C) Immunofluorescence images showing the increased expression and nuclear accumulation of NF-κB (p-p65) in hPDLSCs treated with 10 μg/mL GroEL. The data were derived from three separate experiments ( n=3). NF-κB (p-p65), red; F-actin, green; nuclei, blue. (D) Linear fluorescent quantification of NF-κB (p-p65) in (C) by ImageJ demonstrating the nuclear accumulation of NF-κB (p-p65). The data were derived from three separate experiments ( n=3). (E) Quantitative analysis of total fluorescence density (OD/cell area) showing the increased protein levels of NF-κB (p-p65) in (C). The data are based on 17 cells from three separate experiments ( n=3). *** P<0.001. (F) Immunohistofluorescence images illustrating the enhancement of NF-κB (p-p65) in rat periodontal tissues 4 weeks after the injection of GroEL. NF-κB (p-p65), green; nucleus, blue. The data were derived from four separate experiments ( n=4).

We next explored the changes in signaling pathways, including the MAPK and NF-κB pathways, which mainly participate in the interaction between bacteria and the host [ 34, 35] . By western blot analysis, we detected the activation of NF-κB (p-p65) in hPDLSCs induced by GroEL at 10 μg/mL within 12 h ( Figure 3A). Moreover, GroEL stimulation increased the protein level of p-JNK at 12 h but had no significant influence on p38/MAPK or ERK/MAPK signaling within 12 h ( Figure 3A). Quantitative analysis of these signaling proteins further confirmed that net p-p65 (the ratio of p-p65 to p65) exhibited more significant changes than other signaling proteins ( Figure 3B and Supplementary Figure S6). By immunofluorescence microscopy, we explored the cytoplasmic expression and distribution of p-p65 in hPDLSCs induced by GroEL at 10 μg/mL for 6 h ( Figure 3C). The results showed that GroEL induced an increase in p-p65 in hPDLSCs; moreover, nuclear accumulation of p-p65 in hPDLSCs induced by GroEL was particularly prominent. By linear quantification of fluorescence intensity, we analyzed the nuclear accumulation of p-p65 in hPDLSCs induced by GroEL ( Figure 3D). Using total immunofluorescent quantification of individual hPDLSCs, we confirmed the activation of p-p65 in hPDLSCs induced by GroEL ( Figure 3E). At the tissue level, we detected the expression of p-p65 in the periodontium of rats after injection with GroEL for 4 weeks. The results showed that the expression of p-p65 was greater in the GroEL-treated group than in the control group ( Figure 3F).

### GroEL induces NLRP3 inflammasome via the TLR/NF-κB p-p65 axis

**Figure 4:**
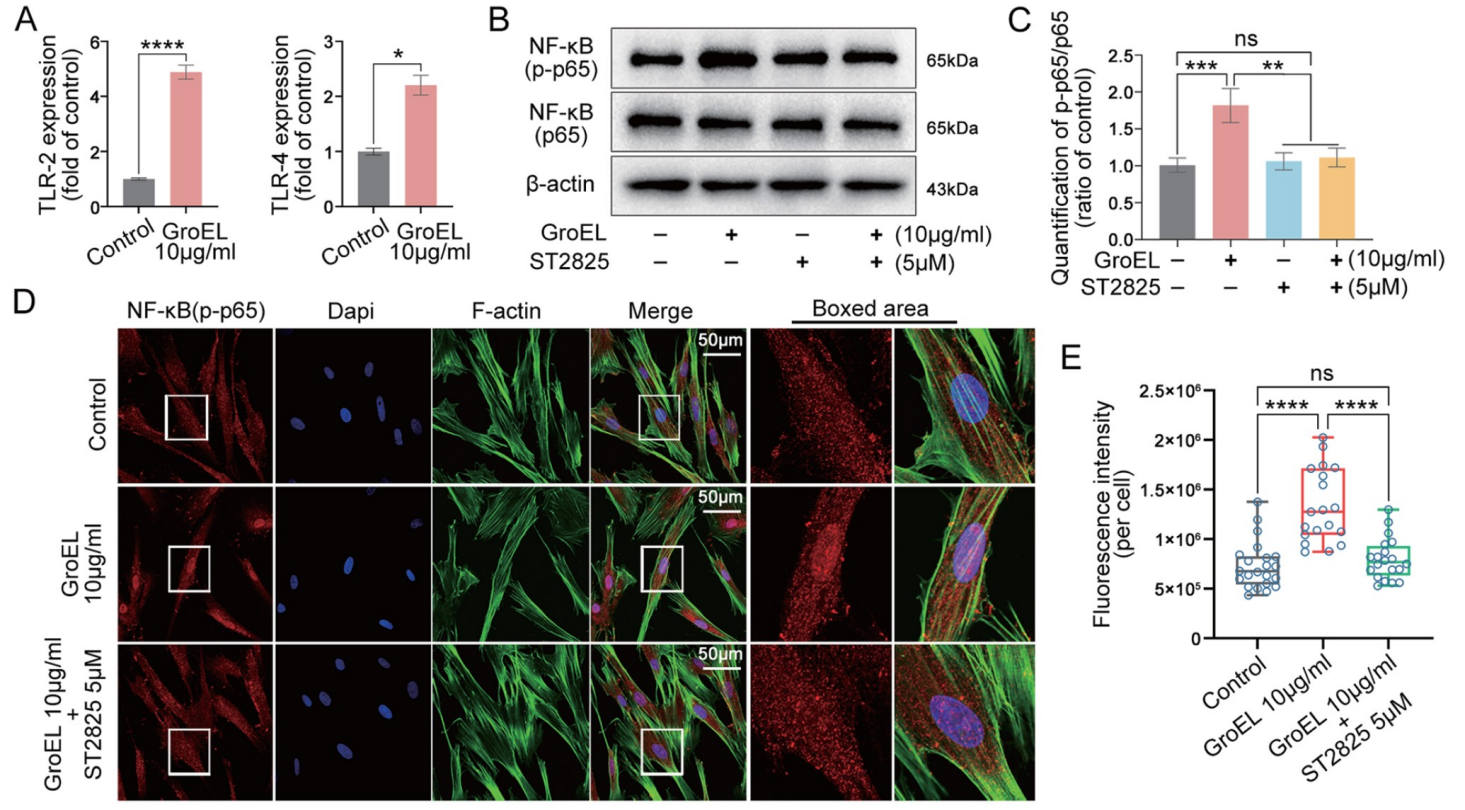
Figure 4 Inhibition of TLRs interferes with GroEL-induced NF-κB signaling (A) qPCR showing the mRNA expressions of TLR-2 and TLR-4 in hPDLSCs induced by GroEL for 2 h. The data were derived from three separate experiments ( n=3). * P<0.05, **** P<0.0001. (B) Western blot images showing the protein expressions of NF-κB (p-p65) and NF-κB (p65) in hPDLSCs induced by GroEL after pretreatment with ST2825. The images were chosen from three separate experiments ( n=3). (C) Quantitative analysis confirming the protein changes in (B). Relative protein expression was normalized to that of β-actin. The data were derived from three separate experiments ( n=3). ** P<0.01, *** P<0.001. (D) Immunofluorescence images showing that ST2825 (5 μM) pretreatment attenuated GroEL-induced activation of NF-κB (p-p65) in hPDLSCs. The images were derived from three separate experiments ( n=3). (E) Quantitative analysis of total fluorescence density (OD/cell area) confirming the changes in the NF-κB (p-p65) protein in (D). The data were analyzed based on 19 cells from three separate experiments ( n=3). **** P<0.0001.

To reveal the underlying mechanism of GroEL-mediated acute inflammation, we first focused on the Toll-like receptors (TLRs), important transmembrane proteins involved in cellular innate immunity [36]. Significant changes of TLR2 and TLR4 were detected in hPDLSCs treated with 10 μg/mL of GroEL for 2 h ( Figure 4A) or 12 h ( Supplementary Figure S7). To determine whether GroEL activates NF-κB signaling through TLR2 and TLR4, we used the inhibitor ST2825 which can specifically inhibit the dimerization of major myeloid differentiation response gene 88 (MyD88), a vital adaptor protein downstream of TLR2 and TLR4 [37]. CCK8 assay showed that 5 μM of ST2825 had no obvious cytotoxic effect on hPDLSCs after 24 h of treatment ( Supplementary Figure S8A). We found that pretreatment with ST2825 significantly reduced the increase in NF-κB p-p65 in hPDLSCs activated by GroEL ( Figure 4B). Quantitative analysis of net p-p65 further confirmed the inhibitory effect of ST2825 on the GroEL-induced increase in p-p65 ( Figure 4C). By immunofluorescence microscopy, we detected changes in the cytoplasmic expression of p-p65 in hPDLSCs induced by GroEL in the presence of ST2825 ( Figure 4D). The results indicated that pretreatment with ST2825 decreased the expression of p-p65 in hPDLSCs induced by GroEL, particularly in the nuclear region. From the total immunofluorescence quantification of individual hPDLSCs, we further confirmed the inhibitory effect of ST2825 on the GroEL-induced increase in p-p65 expression ( Figure 4E). Collectively, these results indicate that GroEL-mediated activation of NF-κB signaling requires the participation of TLR2 and TLR4.

**Figure 5:**
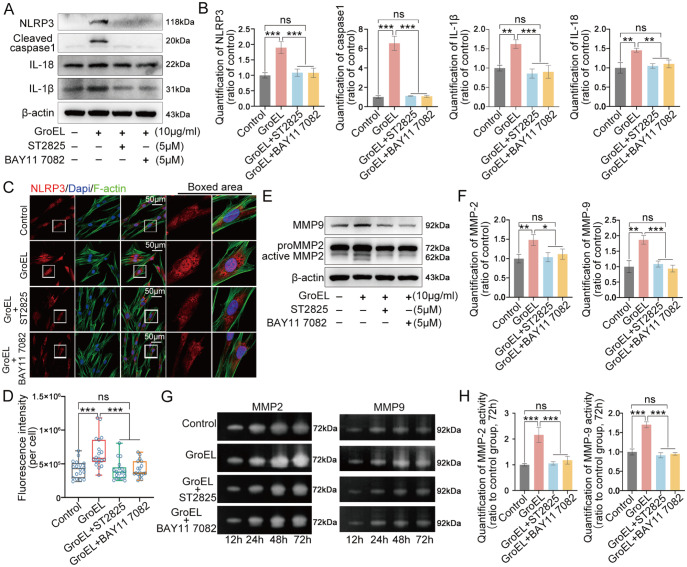
Figure 5 Inhibition of TLRs and NF-κB signalling impairs GroEL-induced activation of the NLRP3 inflammasome and gelatinases (A) Western blot images showing that both ST2825 (5 μM) and BAY11 7082 (5 μM) inhibited GroEL-induced protein expressions of NLRP3, cleaved caspase-1, IL-18 and IL-1β. The images were derived from three separate experiments ( n=3). (B) Quantitative analysis confirming the protein changes in (A). Relative protein expression was normalized to that of β-actin. The data were derived from three separate experiments (n=3). ** P<0.01, *** P<0.001. (C) Immunofluorescence images showing that both ST2825 (5 μM) and BAY11 7082 (5 μM) attenuated GroEL-induced expression of NLRP3 in hPDLSCs. The images were derived from three separate experiments ( n=3). (D) Quantitative analysis of total fluorescence density (OD/cell area) confirming the changes in the level of the NLRP3 protein shown in (C). The data were analyzed based on 17 cells from three separate experiments ( n=3). **** P<0.0001. (E) Western blot images showing that both ST2825 and BAY11 7082 inhibited GroEL-induced MMP-2 and MMP-9 expressions. The images were derived from three separate experiments ( n=3). (F) Quantitative analysis confirming the protein changes in (E). Relative protein expression was normalized to that of β-actin. The data were derived from three separate experiments ( n=3). * P<0.05, ** P<0.01, *** P<0.001. (G) Gelatin zymography images showing that ST2825 and BAY11 7082 both inhibited GroEL-induced activities of MMP-2 and MMP-9 in hPDLSCs. The images were derived from three separate experiments ( n=3). (H) Quantitative analysis confirming the changes in the activities of MMP-2 and MMP-9 at 72 h in (G). The data were derived from three separate experiments ( n=3). *** P<0.001.

To further explore whether GroEL mediates the NLRP3 inflammasome through the TLR/NF-κB p-p65 axis, we detected the changes in the NLRP3 inflammasome and gelatinase activities. For the NLRP3 inflammasome, we performed western blot analysis and found that ST2825 could significantly reduce the expressions of NLRP3 and cleaved caspase 1 in hPDLSCs induced by GroEL ( Figure 5A, upper two lanes); similarly, ST2825 decreased the expressions of IL-1β and IL-18, the downstream effector molecules of the NLRP3 inflammasome ( Figure 5A, middle two lanes).

To confirm the role of the downstream part of the TLR/NF-κB p-p65 axis, we used BAY11-7082, a well-recognized inhibitor of NF-κB signaling [32]. The inhibitory role of BAY11-7082 is shown in Supplementary Figure S9. CCK8 assay showed that 5 μM of BAY11-7082 had no obvious cytotoxic effect on hPDLSCs after 24 h of treatment ( Supplementary Figure S8B). We found that inhibition of NF-κB p-p65 significantly reduced the expressions of NLRP3, cleaved caspase 1, IL-1β and IL-18 in hPDLSCs induced by GroEL ( Figure 5A, last column). Quantitative analysis further confirmed the changes in these proteins ( Figure 5B). We then detected the cytoplasmic expression and distribution of NLRP3 in hPDLSCs induced by GroEL in the presence of ST2825 or BAY11-7082, and the results showed that ST2825 or BAY11-7082 could greatly impair the enhancement of NLRP3 in hPDLSCs induced by GroEL, especially in the nuclear region ( Figure 5C). From the total immunofluorescent quantification of individual hPDLSCs, we further confirmed the inhibitory effect of ST2825 or BAY11-7082 on the GroEL-induced increase in the expression of NLRP3 ( Figure 5D). For gelatinases, western blot analysis showed that ST2825 could decrease the expressions of MMP-2 and MMP-9 in hPDLSCs induced by GroEL, similar to BAY11-7082 ( Figure 5E). Quantitative analyses further confirmed the changes in MMP-2 and MMP-9 in hPDLSCs induced by GroEL in the presence of ST2825 or BAY11-7082 ( Figure 5F). We finally used gelatin zymography to detect changes in the activities of MMP-2 and MMP-9. The results showed that ST2825 or BAY11-7082 could reduce the ability of active MMP-2 (left) and MMP-9 (right) to hydrolyze gelatin ( Figure 5G). Quantitative analysis confirmed the changes in the activity of MMP-2 and MMP-9 in hPDLSCs induced by GroEL in the presence of ST2825 or BAY11-7082 ( Figure 5H).

Collectively, the data provided in Figures 4 and 5 indicate that GroEL promotes the NLRP3 inflammasome via the TLR/NF-κB p-p65 axis.

### GroEL induces the destruction of periodontal tissue and alveolar bone loss in a rat model

**Figure 6:**
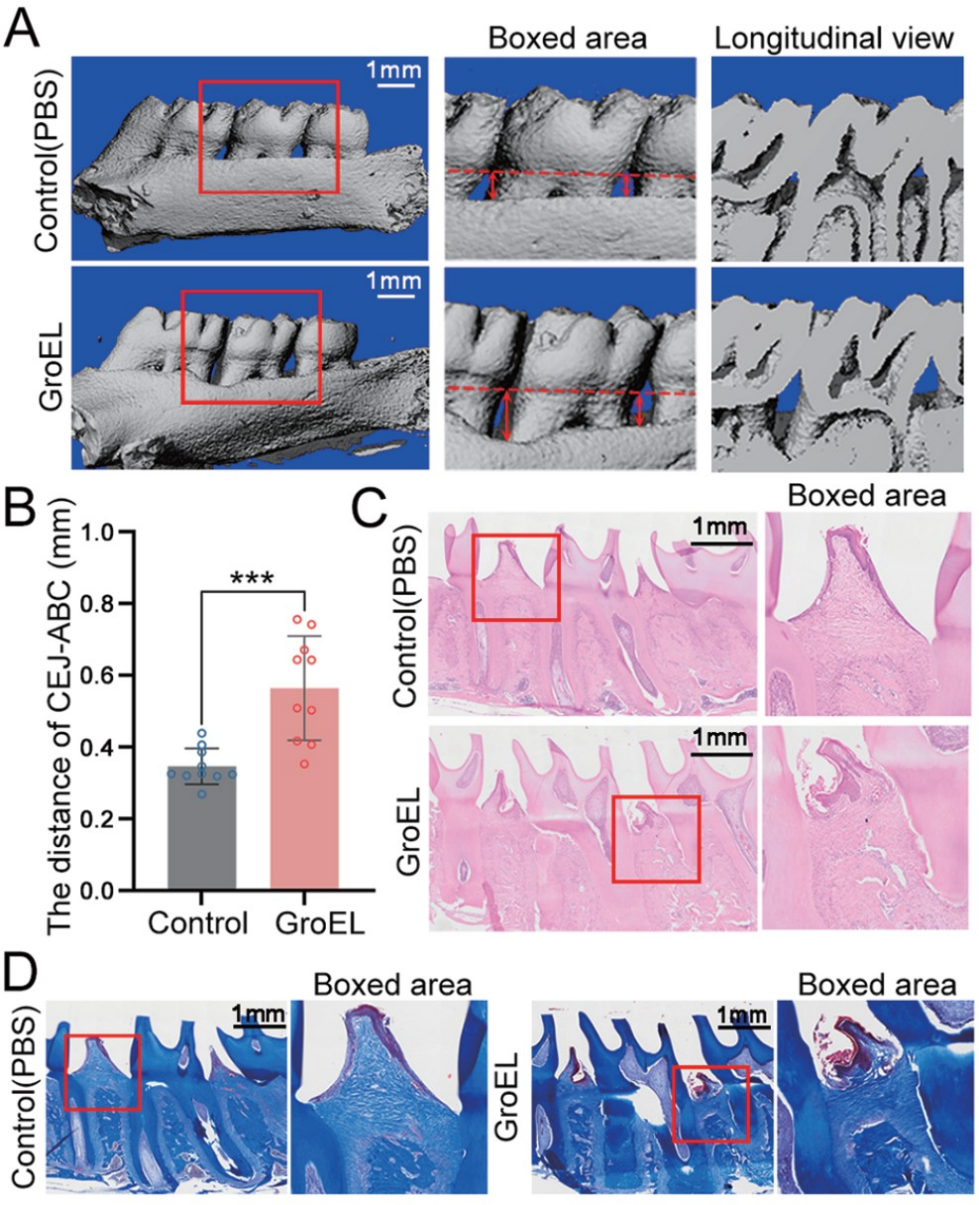
Figure 6 GroEL induces the destruction of periodontal tissue and alveolar bone loss (A) Micro-CT images of the rat maxilla showing alveolar bone absorption in rats treated with GroEL for 4 weeks. Red arrows represent the distance from the CEJ to the ABC. The results were based on five separate experiments ( n=5). (B) Quantitative analysis of the distance travelled by the CEJ-ABC in (A). The data were analyzed based on 10 measurements from five separate experiments ( n=5). *** P<0.001. (C) Histological images of H&E staining showing periodontal tissue damage in GroEL-stimulated rats. The images are based on five separate experiments ( n=5). (D) Histological images of Masson staining showing periodontal tissue damage in rat periodontal tissues 4 weeks after the injection of GroEL. The images are based on five separate experiments ( n=5).

To further investigate the inflammatory effect of GroEL, we analyzed the entire periodontium-alveolar bone area ( Figure 6). 3D μ-CT reconstructions revealed that GroEL administration led to alveolar bone loss ( Figure 6A). The distance between the cemento-enamel junction (CEJ) and alveolar bone crest (ABC) was significantly greater in the GroEL-treated group than in the control (PBS) group ( Figure 6B). H&E and Masson staining demonstrated that the destruction of periodontal tissue and alveolar bone in GroEL-treated rats was more extensive than that in PBS-treated rats ( Figure 6C,D). Taken together, these *in vivo* experiments indicated that GroEL treatment induces the destruction of periodontal tissue and alveolar bone loss.

## Discussion

**Figure 7:**
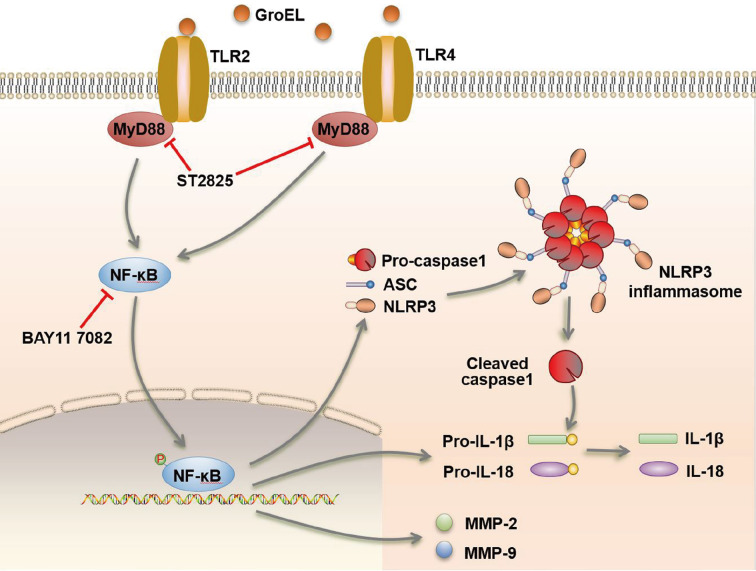
Figure 7 Schematic of GroEL-mediated activation of the NLRP3 inflammasome in hPDLSCs through the TLR-NF-κB signalling axis GroEL stimulates hPDLSCs via TLR2 and TLR4, activates the cytoplasmic NLRP3 inflammasome and its downstream inflammatory factors, IL-1β and IL18, and enhances the activity of gelatinases. These cytoplasmic cascade events involve NF-κB signaling.

Pathogenic bacteria release a variety of microbial virulence factors, such as microbial peptides, LPS, lipoteichoic acid and cytolethal distending toxin (CDT), which act on periodontal cells and induce inflammation [ 38‒ 40] . GroEL is an important virulence factor implicated in bacteria-host interaction and may be involved in the progression of periodontitis. *P*. *gingivalis* GroEL upregulates inflammatory cytokines (IL-6 and IL-8) in osteoblasts and PDLCs through NF-κB signaling and promotes alveolar bone loss in rats [ 18, 20] . The NLRP3 inflammasome is essential for periodontitis pathogenesis. Defective activation of the NLRP3 inflammasome in periodontal cells, such as osteoblasts, PDLFs, and macrophages, leads to cellular dysfunction and further contributes to the disorder of periodontal tissues [41]. This study investigated the impact of GroEL on the NLRP3 inflammasome in hPDLSCs both *in vitro* and *in vivo*. Our results showed that GroEL activates the cytoplasmic NLRP3 inflammasome and its downstream inflammatory factors IL-1β and IL18, and enhances the activity of gelatinases in hPDLSCs through the TLR-NF-κB signaling axis ( Figure 7).

The NLRP3 inflammasome, known as a cytosolic multiprotein complex, reacts to external stimuli, endogenous danger signals, and microbial infection [42]. Studies have shown that a variety of pathogens associated with periodontitis can cause NLRP3 inflammasome activation and inflammatory responses [43]. In addition to pathogenic pathogens, microbial virulence factors can also induce inflammatory responses associated with NLRP3 inflammasome signaling [ 38, 44] . *A*. *actinomycetemcomitans* LtxA promotes NLRP3 inflammasome activation and intercellular communication, inducing inflammatory cell death in THP-1 cells [27]. Assembled NLRP3 inflammasome activates caspase-1 to cleave pro-IL-1β and pro-IL-18, promoting the release of IL-1β and IL-18 [42]. Inflammatory cytokine IL-1β can promote osteoclastogenesis by upregulating the production of MMPs and cathepsin K in PDLFs and PDLSCs [ 45, 46] . In this study, we found that local injection with GroEL in the distal and mesial interdental gingiva of the maxillary second molar induces the destruction of periodontal tissue and alveolar bone loss, which suggests that GroEL is involved in the progression of periodontitis. We found that GroEL activated the NLRP3 inflammasome and upregulated its downstream inflammatory factors IL-1β and IL-18 in hPDLSCs *in vitro*, and local injection with GroEL in rats increased the expressions of NLRP3, IL-1β and IL-18 in periodontal tissue in the GroEL-treated group. In addition, GroEL also increased the expressions of inflammatory factors (IL-6 and TNFα) in hPDLSCs both *in vitro* and *in vivo*. These findings demonstrate that GroEL has the ability to trigger an inflammatory response in periodontitis.

MMPs are significant enzymes involved in the breakdown of the extracellular matrix (ECM), which regulates bone resorption [ 47, 48] . The gelatinases MMP-2 and MMP-9 are crucial for breaking down proteins in ECM materials, such as the bone matrix. MMP-2 can both directly and indirectly accelerate the degradation of type I collagen, which is the most abundant collagen fiber in the periodontal ligament [ 32, 49] . It has been confirmed that MMP-9 is involved in the invasive activity of osteoclasts [50]. The activity of MMPs is regulated by endogenous inhibitors, such as TIMPs. There are four distinct TIMPs known to exist: TIMP1, TIMP2, TIMP3, and TIMP4. Furthermore, MMP2 and MMP9 are naturally inhibited by TIMP2 and TIMP1, respectively [ 51, 52] . Behm *et al*. [46] reported that IL-1β regulated the expressions of MMP-1, MMP-2 and TIMP-1 in force-treated PDLSCs. Moreover, Peng *et al*. [53] reported that the suppression of MMP-2 and MMP-9 activity by scutellarin was reversed by NLRP3 overexpression in lung epithelial cells. In this study, we investigated the effects of GroEL on gelatinases in hPDLSCs and found that GroEL increased the expression and activity of gelatinases in hPDLSCs, and at the tissue level, GroEL induced higher expressions of MMP-2 and MMP-9 in the rat periodontium compared to that in the non-treated control group. The changes in the expressions of *TIMP-1* and *TIMP-2* were confirmed by qPCR, which revealed that GroEL downregulates the gene expressions of *TIMP-1* and *TIMP-2*. Overall, these findings suggested that GroEL has a great impact on ECM degradation via the upregulation of MMP-2 and MMP-9, which may contribute to the destruction of periodontal tissue.

TLRs are important pattern recognition receptors (PRRs) associated with the identification of periodontal pathogens and the induction of the host innate immune response [54]. Previous studies reported that TLR2 and TLR4 might be involved in the recognition of GroEL and mediate downstream signaling [ 21, 29] . A previous study reported that *P*. *gingivalis* GroEL stimulated the NF-κB signaling pathway via TLR2 and TLR4 in THP-1 cells, and anti-hTLR2 and anti-hTLR4 antibodies clearly decreased NF-κB transcriptional activity [55]. Our previous study verified that GroEL treatment upregulated TLR2 and TLR4 expressions [29], which is consistent with the results of the present study. Moreover, some studies showed that NLRP3 inflammasome activation upregulates the expression levels of NLRP3 and pro-IL-1β through TLR/NF-κB-mediated signaling [ 56, 57] . MAPKs are important molecules that function upstream of numerous proinflammatory pathways [58]. Waetzig *et al*. [59] reported that inhibition of JNK/MAPK decreased the LPS-induced expressions of proinflammatory factors, such as cyclooxygenase-2 (Cox-2), TNFα, MCP-1 and IL-6, in microglia, while ERK/MAPK and p38 had a more noticeable impact on LPS-induced cellular enlargement. The involvement of p38/MAPK in regulating the activation of the NLRP inflammasome has been reported but is controversial. Dong *et al*. [60] demonstrated that peptidyl-prolyl cis-trans isomerase (Pin1) regulates the LPS-activated NLRP3 inflammasome by activating the p38/MAPK pathway in macrophages. However, Shin *et al*. [61] showed that p38/MAPK adversely regulates the NLRP3 inflammasome in macrophages by controlling Ca ^2+^ mobilization and that hyperactivation of the NLRP3 inflammasome in p38-deficient cells is unaffected by ERK or JNK. In this study, we used western blot analysis to investigate the links between the NF-κB and MAPK signaling pathways and TLR signaling pathways, and found that GroEL treatment had no significant influence on p38/MAPK or ERK/MAPK signaling, and that the protein level of p-JNK was increased after 12 h of GroEL treatment, while the NF-κB (p-p65) level was significantly increased within 12 h. The change in NF-κB signaling was more significant than the changes in the JNK/MAPK signaling pathway, so we focused on NF-κB signaling. In addition, at the tissue level, the expression of p-p65 was increased in the GroEL-treated group. Afterward, ST2825, which can specifically inhibit MyD88 dimerization, was used to interrupt TLR signaling. Our results showed that ST2825 pretreatment attenuated GroEL-induced NF-κB (p-p65) expression and nuclear translocation in hPDLSCs, suggesting that GroEL activates NF-κB signaling through TLR2 and TLR4. BAY11-7082 (an NF-κB inhibitor) inhibited GroEL-induced NF-κB signaling. Pretreatment with ST2825 and BAY11-7082 can antagonize the functions of GroEL. The results showed that ST2825 and BAY11 7082 attenuated GroEL-upregulated expressions of NLRP3, cleaved caspase 1, IL-1β and IL-18, and attenuated gelatinase activity in hPDLSCs, suggesting that GroEL mediates the NLRP3 inflammasome through the TLR/NF-κB p-p65 axis.

In summary, this study demonstrated that local injection with GroEL in rats induces the destruction of periodontal tissue and alveolar bone loss. GroEL activates the NLRP3 inflammasome and upregulates the activities of inflammatory factors, such as IL-1β, IL-18, IL-6 and TNFα, and gelatinases both *in vitro* and *in vivo*. Inhibition of TLRs interrupts GroEL-induced NF-κB signaling, and upregulation of the TLR-NF-κB signaling axis is probably involved in GroEL-induced NLRP3 inflammasome activation and inflammatory responses in hPDLSCs. Our findings may increase our understanding that GroEL, a virulence factor, may be involved in the progression of periodontitis.

## Supporting information

24028Supplementary_materials

## Supplementary Data

Supplementary data is available at *Acta Biochimica et Biophysica Sinica* online.

## References

[1] Hajishengallis G (2015). Periodontitis: From microbial immune subversion to systemic inflammation. Nat Rev Immunol.

[2] Pihlstrom BL, Michalowicz BS, Johnson NW (2005). Periodontal diseases. Lancet.

[3] Tonetti MS, Jepsen S, Jin L, Otomo-Corgel J (2017). Impact of the global burden of periodontal diseases on health, nutrition and wellbeing of mankind: a call for global action. J Clinic Periodontol.

[4] Kapila YL (2021). Oral health’s inextricable connection to systemic health: special populations bring to bear multimodal relationships and factors connecting periodontal disease to systemic diseases and conditions. Periodontol 2000.

[5] Nwizu N, Wactawski-Wende J, Genco RJ (2020). Periodontal disease and cancer: epidemiologic studies and possible mechanisms. Periodontol 2000.

[6] Hasturk H, Kantarci A (2015). Activation and resolution of periodontal inflammation and its systemic impact. Periodontol 2000.

[7] Zhang S, Yu N, Arce RM (2020). Periodontal inflammation: integrating genes and dysbiosis. Periodontol 2000.

[8] Hajishengallis G, Lamont RJ, Koo H (2023). Oral polymicrobial communities: assembly, function, and impact on diseases. Cell Host Microbe.

[9] Hayer-Hartl M, Bracher A, Hartl FU (2016). The GroEL-GroES chaperonin machine: a nano-cage for protein folding. Trends Biochem Sci.

[10] Hartl FU, Bracher A, Hayer-Hartl M (2011). Molecular chaperones in protein folding and proteostasis. Nature.

[11] Jung YJ, Choi YJ, An SJ, Lee HR, Jun HK, Choi BK (2017). Tannerella forsythia GroEL induces inflammatory bone resorption and synergizes with interleukin-17. Mol Oral Microbiol.

[12] Gholizadeh P, Pormohammad A, Eslami H, Shokouhi B, Fakhrzadeh V, Kafil HS (2017). Oral pathogenesis of
*Aggregatibacter actinomycetemcomitans*. Microb Pathog.

[13] Zhu D, Fan Y, Wang X, Li P, Huang Y, Jiao J, Zhao C (2023). Characterization of molecular chaperone GroEL as a potential virulence factor in cronobacter sakazakii. Foods.

[14] Zhang L, Cui Y, Yang Y, Wei J, Liu W, Cai L, Wang L (2021). The virulence factor GroEL promotes gelatinase secretion from cells in the osteoblast lineage: implication for direct crosstalk between bacteria and adult cells. Arch Oral Biol.

[15] Fukui M, Hinode D, Yokoyama M, Tanabe S, Yoshioka M (2006). Salivary immunoglobulin A directed to oral microbial GroEL in patients with periodontitis and their potential protective role. Oral Microbiol Immunol.

[16] Leishman SJ, Ford PJ, Do HL, Palmer JE, Heng NCK, West MJ, Seymour GJ (2012). Periodontal pathogen load and increased antibody response to heat shock protein 60 in patients with cardiovascular disease. J Clinic Periodontol.

[17] Yamazaki K, Ueki-maruayama K, Honda T, Nakajima T, Seymour GJ (2004). Effect of periodontal treatment on the serum antibody levels to heat shock proteins. Clin Exp Immunol.

[18] Lin FY, Hsiao FP, Huang CY, Shih CM, Tsao NW, Tsai CS, Yang SF (2014). Porphyromonas gingivalis GroEL induces osteoclastogenesis of periodontal ligament cells and enhances alveolar bone resorption in rats. PLoS One.

[19] Hinode D, Yoshioka M, Tanabe SI, Miki O, Masuda K, Nakamura R (1998). The GroEL-like protein from
*Campylobacter rectus*: immunological characterization and interleukin-6 and -8 induction in human gingival fibroblast. FEMS Microbiol Lett.

[20] Lin HH, Lin YW, Wu CY, Hsiao FP, Lai YL, Hung SL (2021). Stimulatory effects of
*Porphyromonas gingivalis* GroEL protein on interleukin-6 and interleukin-8 in human osteoblasts. J Formos Med Assoc.

[21] Lee H, Jun H, Kim H, Lee S, Choi B (2012). *Fusobacterium nucleatum* GroEL induces risk factors of atherosclerosis in human microvascular endothelial cells and ApoE
^−/−^ mice. Mol Oral Microbiol.

[22] Fu J, Wu H (2023). Structural mechanisms of NLRP3 inflammasome assembly and activation. Annu Rev Immunol.

[23] Isola G, Polizzi A, Santonocito S, Alibrandi A, Williams RC (2022). Periodontitis activates the NLRP3 inflammasome in serum and saliva. J Periodontol.

[24] Olsen I, Yilmaz Ö (2016). Modulation of inflammasome activity by
*Porphyromonas gingivalis* in periodontitis and associated systemic diseases. J Oral Microbiol.

[25] Guan Y, Gu Y, Li H, Liang B, Han C, Zhang Y, Liu Q (2022). NLRP3 inflammasome activation mechanism and its role in autoimmune liver disease. Acta Biochim Biophys Sin.

[26] Chen Y, Yang Q, Lv C, Chen Y, Zhao W, Li W, Chen H (2021). NLRP3 regulates alveolar bone loss in ligature-induced periodontitis by promoting osteoclastic differentiation. Cell Prolif.

[27] Kelk P, Moghbel NS, Hirschfeld J, Johansson A (2022). Aggregatibacter actinomycetemcomitans leukotoxin activates the NLRP3 inflammasome and cell-to-cell communication. Pathogens.

[28] Lu WL, Song DZ, Yue JL, Wang TT, Zhou XD, Zhang P, Zhang L (2017). NLRP 3 inflammasome may regulate inflammatory response of human periodontal ligament fibroblasts in an apoptosis-associated speck-like protein containing aCARD (ASC)-dependent manner. Int Endod J.

[29] Zhang L, Cheng L, Cui Y, Wu Z, Cai L, Yang L, Duan M (2021). The virulence factor GroEL directs the osteogenic and adipogenic differentiation of human periodontal ligament stem cells through the involvement of JNK/MAPK and NF-κB signaling. J Periodontol.

[30] Zhou C, Yang Y, Duan M, Chen C, Pi C, Zhang D, Liu X (2023). Biomimetic fibers based on equidistant micropillar arrays determines chondrocyte fate via mechanoadaptability. Adv Healthcare Mater.

[31] Liu Y, Pu X, Duan M, Chen C, Zhao Y, Zhang D, Xie J (2023). Biomimetic fibers derived from an equidistant micropillar platform dictate osteocyte fate via mechanoreception. Nano Lett.

[32] Xie J, Wang CL, Yang W, Wang J, Chen C, Zheng L, Sung KLP (2018). Modulation of MMP-2 and MMP-9 through connected pathways and growth factors is critical for extracellular matrix balance of intra-articular ligaments. J Tissue Eng Regen Med.

[33] Nagase H, Visse R, Murphy G (2006). Structure and function of matrix metalloproteinases and TIMPs. Cardiovasc Res.

[34] Nagahama M, Shibutani M, Seike S, Yonezaki M, Takagishi T, Oda M, Kobayashi K (2013). The p38 MAPK and JNK pathways protect host cells against clostridium perfringens beta-toxin. Infect Immun.

[35] Chen S, Wu Z, He Y, Zhu L, Wang J, Lin H, Xie J (2023). Cyclic di-adenosine monophosphate regulates the osteogenic and adipogenic differentiation of hPDLSCs via MAPK and NF-&kappa;B signaling. Acta Biochim Biophys Sin.

[36] Fitzgerald KA, Kagan JC (2020). Toll-like receptors and the control of immunity. Cell.

[37] Zhang X, Wan Y, Feng J, Li M, Jiang Z (2021). Involvement of TLR2/4‑MyD88‑NF‑κB signaling pathway in the pathogenesis of intracranial aneurysm. Mol Med Rep.

[38] Tiranathanagul S, Yongchaitrakul T, Pattamapun K, Pavasant P (2004). *Actinobacillus actinomycetemcomitans* lipopolysaccharide activates matrix metalloproteinase-2 and increases receptor activator of nuclear factor-kappaB ligand expression in human periodontal ligament cells. J Periodontol.

[39] Zhu C, Zhou J, Li T, Mu J, Jin L, Li S (2020). Urocortin participates in LPS-induced apoptosis of THP-1 macrophages via S1P-cPLA2 signaling pathway. Eur J Pharmacol.

[40] Jones KJ, Ekhlassi S, Montufar-Solis D, Klein JR, Schaefer JS (2010). Differential cytokine patterns in mouse macrophages and gingival fibroblasts after stimulation with
*Porphyromonas gingivalis* or
*Escherichia coli* lipopolysaccharide. J Periodontol.

[41] Zhao Y, Quan Y, Lei T, Fan L, Ge X, Hu S (2022). The role of inflammasome NLPR3 in the development and therapy of periodontitis. Int J Med Sci.

[42] Huang Y, Xu W, Zhou R (2021). NLRP3 inflammasome activation and cell death. Cell Mol Immunol.

[43] Wang X, Jia Y, Wen L, Mu W, Wu X, Liu T, Liu X (2021). *Porphyromonas gingivalis* promotes colorectal carcinoma by activating the hematopoietic nlrp3 inflammasome. Cancer Res.

[44] Liu S, Du J, Li D, Yang P, Kou Y, Li C, Zhou Q (2020). Oxidative stress induced pyroptosis leads to osteogenic dysfunction of MG63 cells. J Mol Hist.

[45] He Y, Wu Z, Chen S, Wang J, Zhu L, Xie J, Zhou C (2022). Activation of the pattern recognition receptor NOD1 in periodontitis impairs the osteogenic capacity of human periodontal ligament stem cells via p38/MAPK signalling. Cell Prolif.

[46] Behm C, Nemec M, Blufstein A, Schubert M, Rausch-Fan X, Andrukhov O, Jonke E (2021). Interleukin-1β induced matrix metalloproteinase expression in human periodontal ligament-derived mesenchymal stromal cells under in vitro simulated static orthodontic forces. Int J Mol Sci.

[47] Du X, Cai L, Xie J, Zhou X (2023). The role of TGF-beta3 in cartilage development and osteoarthritis. Bone Res.

[48] Chen H, Cui Y, Zhang D, Xie J, Zhou X (2022). The role of fibroblast growth factor 8 in cartilage development and disease. J Cell Mol Med.

[49] Hudson DM, Garibov M, Dixon DR, Popowics T, Eyre DR (2017). Distinct post-translational features of type I collagen are conserved in mouse and human periodontal ligament. J Periodontal Res.

[50] Delaissé JM, Engsig MT, Everts V, del Carmen Ovejero M, Ferreras M, Lund L, Vu TH (2000). Proteinases in bone resorption: obvious and less obvious roles. Clin Chim Acta.

[51] Xie J, Fu N, Cai LY, Gong T, Li G, Peng Q, Cai XX (2015). The effects of interleukin-1β in modulating osteoclast-conditioned medium’s influence on gelatinases in chondrocytes through mitogen-activated protein kinases. Int J Oral Sci.

[52] Murphy G (2011). Tissue inhibitors of metalloproteinases. Genome Biol.

[53] Peng L, Wen L, Shi QF, Gao F, Huang B, Meng J, Hu CP (2020). Scutellarin ameliorates pulmonary fibrosis through inhibiting NF-κB/NLRP3-mediated epithelial–mesenchymal transition and inflammation. Cell Death Dis.

[54] Kawai T, Akira S (2010). The role of pattern-recognition receptors in innate immunity: update on Toll-like receptors. Nat Immunol.

[55] Argueta JGM, Shiota S, Yamaguchi N, Masuhiro Y, Hanazawa S (2006). Induction of
*Porphyromonas gingivalis* GroEL signaling via binding to Toll‐like receptors 2 and 4. Oral Microbiol Immunol.

[56] Bauernfeind FG, Horvath G, Stutz A, Alnemri ES, MacDonald K, Speert D, Fernandes-Alnemri T (2009). Cutting edge: NF-κB activating pattern recognition and cytokine receptors license NLRP3 inflammasome activation by regulating NLRP3 expression. J Immunol.

[57] Wang L, Jin H, Ye D, Wang J, Ao X, Dong M, Niu W (2016). Enterococcus faecalis lipoteichoic acid-induced NLRP3 inflammasome via the activation of the nuclear factor kappa B pathway. J Endods.

[58] Arthur JSC, Ley SC (2013). Mitogen-activated protein kinases in innate immunity. Nat Rev Immunol.

[59] Waetzig V, Czeloth K, Hidding U, Mielke K, Kanzow M, Brecht S, Goetz M (2005). c-Jun N-terminal kinases (JNKs) mediate pro-inflammatory actions of microglia. Glia.

[60] Dong R, Xue Z, Fan G, Zhang N, Wang C, Li G, Da Y (2021). Pin1 promotes NLRP3 inflammasome activation by phosphorylation of p38 MAPK pathway in septic shock. Front Immunol.

[61] Shin JN, Rao L, Sha Y, Abdel Fattah E, Hyser J, Eissa NT (2021). p38 MAPK activity is required to prevent hyperactivation of NLRP3 inflammasome. J Immunol.

