# Supplementary material for: GroEL triggers NLRP3 inflammasome activation through the TLR/NF-κB p-p65 axis in human periodontal ligament stem cells: GroEL triggers NLRP3 inflammasome activation
Source: Acta Biochim Biophys Sin (Shanghai). 2024 Apr 10;56(9):1340–51. doi: 10.3724/abbs.2024050 (PMC11532219; doi:10.3724/abbs.2024050)
Supplement: 24028Supplementary_materials [file 24028Supplementary_materials.pdf]

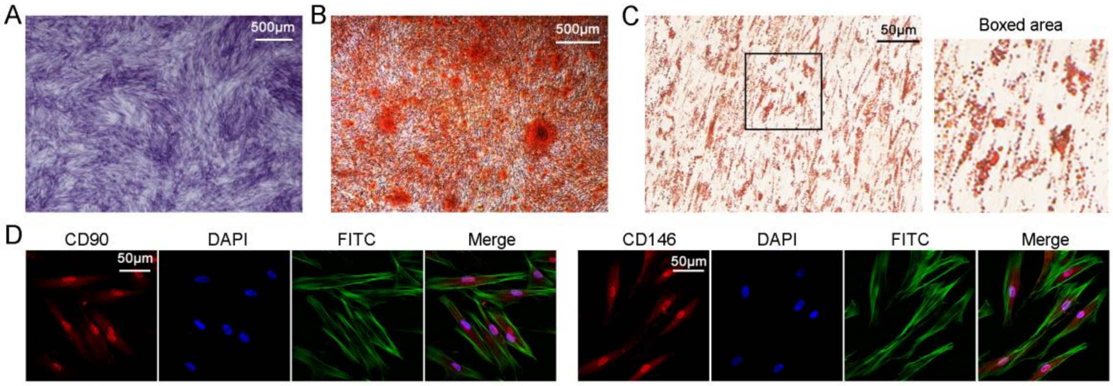

Supplementary Figure S1. The stemness characterization of hPDLSCs (A,B) The osteogenic differentiation capacity of hPDLSCs was evaluated using ALP staining (A) and Alizarin Red staining (B). (C) The adipogenesis capacity of hPDLSCs was evaluated using Oil Red O staining. (D) Immunofluorescence images showing the positive expression of CD90 and CD146 in harvested hPDLSCs. The data were derived from three independent experiments (*n*=3). CD90 and CD146, red; F-actin, green; nucleus, blue.

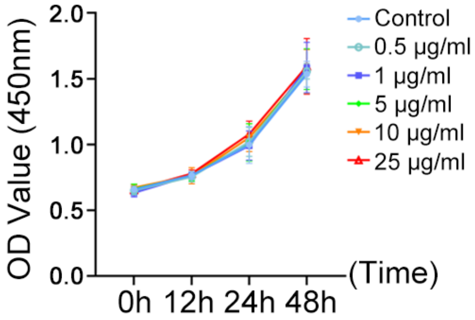

Supplementary Figure S2. Effects of GroEL on the cell viability of hPDLSCs Cell Counting Kit-8 (CCK8) assay showing the cytotoxicity of the GroEL on hPDLSCs. Different concentrations (0, 0.5, 1, 5, 10 and 25 μg/mL) of GroEL showed no significant impact on the cell viability of hPDLSCs.

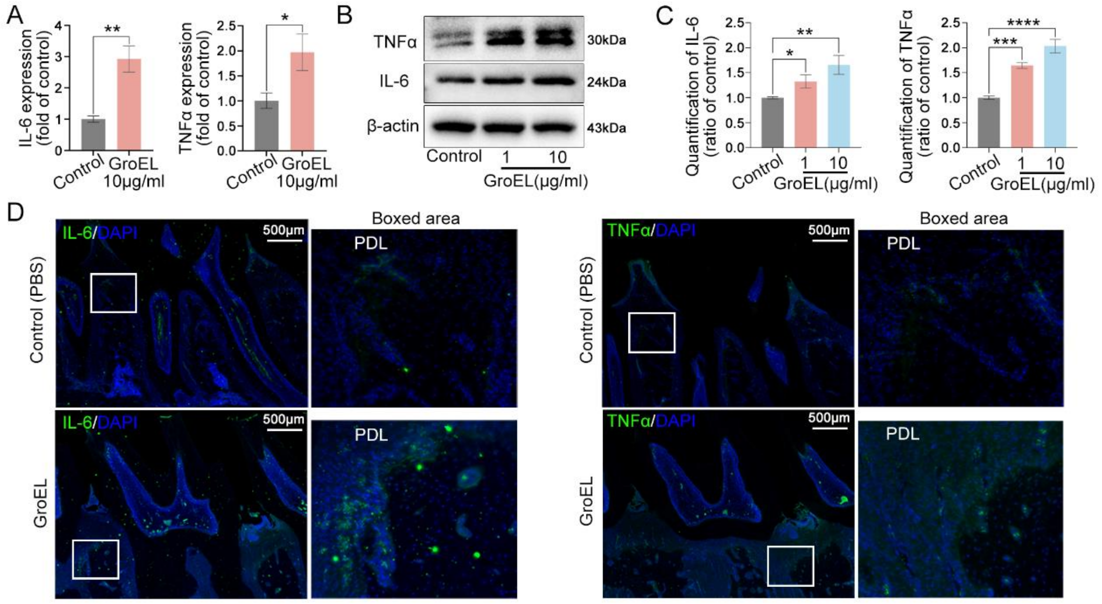

Supplementary Figure S3. GroEL induces the expressions of IL-6 and TNF-α in hPDLSCs (A) qPCR showing the mRNA expressions of IL-6 and TNF-α in hPDLSCs induced by GroEL. The data were derived from three independent experiments (*n*=3). ***P<0.001, ****P<0.0001. (B) Western blot images showing protein expressions of IL-6 and TNF-α in hPDLSCs treated with GroEL. The images are representative of three independent experiments (*n*=3). (C) Quantitative analysis confirming the protein changes in (B). Relative protein expressions were normalized with β-actin. The data were derived from three independent experiments (*n*=3). **P<0.01, ****P<0.0001. (D) Immunofluorescence images showing the increased expressions of IL-6 and TNF-α in rat periodontal tissues post injection with GroEL for 4 weeks. IL-6 and TNF-α, green; nucleus, blue. The data were derived from four independent experiments (*n*=4).

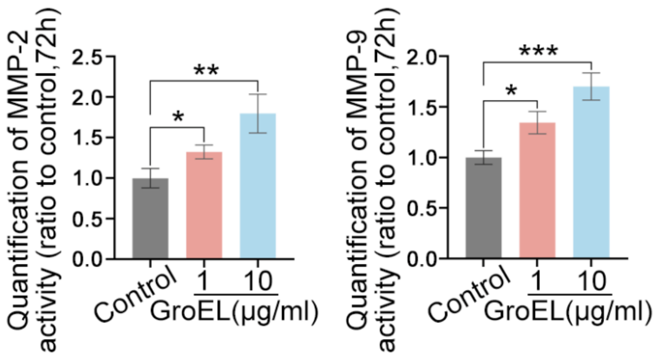

Supplementary Figure S4. Quantitative analysis of the activities of MMP-2 and MMP-9 in hPDLSCs induced by GroEL for 72 h The data were derived from three independent experiments (*n*=3). *P<0.05, **P<0.01, ***P<0.001.

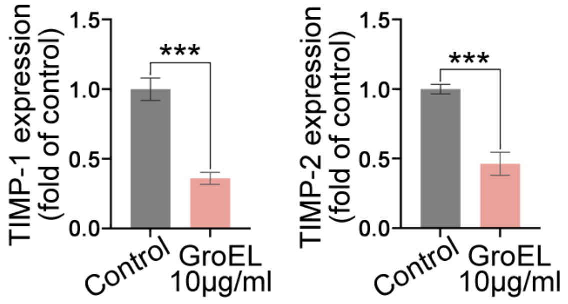

Supplementary Figure S5. qPCR results showing the mRNA expressions of TIMP-1 and TIMP-2 in hPDLSCs induced by GroEL The data were derived from three independent experiments (*n*=3). ***P<0.001.

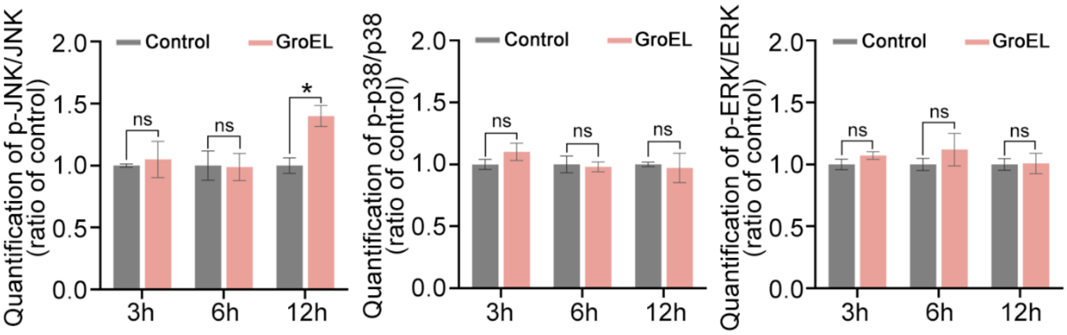

Supplementary Figure S6. Quantitative analysis of p-JNK/JNK, p-p38/p38 and p-Erk1/2/Erk1/2 in Figure 3A The data were derived from three independent experiments (*n*=3). *P<0.05.

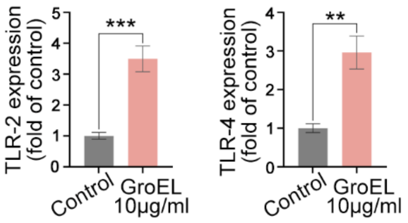

Supplementary Figure S7. Expressions of *TLR2* and *TLR4* mRNA in GroEL-stimulated hPDLSCs qPCR showing the mRNA expressions of *TLR-2* and *TLR-4* in hPDLSCs induced

by GroEL for 12 h. The data were derived from three independent experiments (*n*=3). ***P*<0.01, ****P*<0.001.

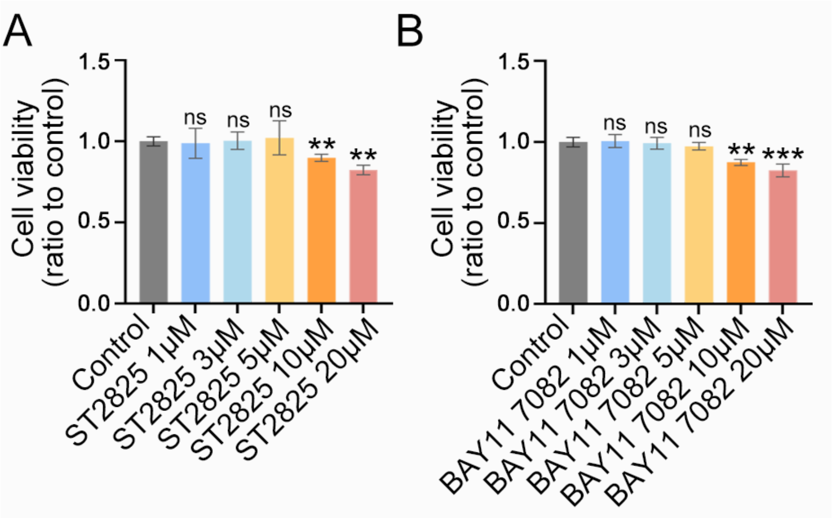

Supplementary Figure S8. Effects of ST2825 and BAY11-7082 on the cell viability of hPDLSCs (A) Cell Counting Kit-8 (CCK8) assay showing the cytotoxicity of ST2825 at different concentrations (0, 1, 3, 5, 10 and 20 μM) on hPDLSCs. The data were derived from three independent experiments (*n*=3). ***P*<0.01. (B) Cell Counting Kit-8 (CCK8) assay showing cytotoxicity of BAY11-7082 at different concentrations (0, 1, 3, 5, 10 and 20 μM) on hPDLSCs. The data were derived from three independent experiments (*n*=3). ***P*<0.01, ****P*<0.001.

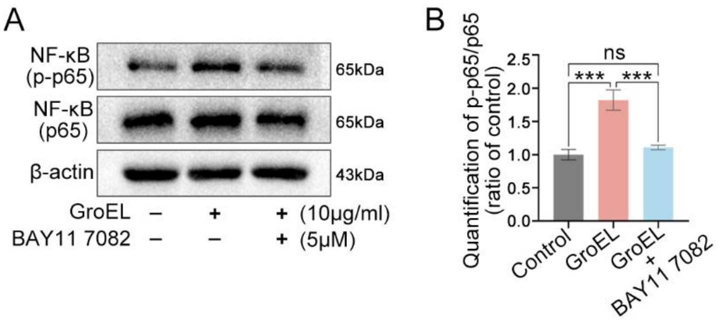

Supplementary Figure S9. The specific inhibitor BAY11 7082 shows a strong inhibitory effect of NF-κB signaling in the presence of GroEL (A) Western blot images showing the protein expression of NF-κB (total p65 and p-p65) in hPDLSCs treated with BAY11-7082 in the presence of GroEL. The images are representative of three independent experiments (*n*=3). (B)

Quantitative analysis confirming the protein changes in (A). Relative protein expressions were normalized with β-actin. The data were derived from three independent experiments (*n*=3). ***P<0.001.

Supplementary Table S1. Sequences of primer pairs used in the study

Protein name	Gene name/gene ID	Primer sequence (5′→3′)
Glyceraldehyde-3-phosphate dehydrogenase	*GAPDH* (NM_001256799.3)	Forward: GACAGTCAGCCGCATCTTCT<br>Reverse: GCGCCCAATACGACCAAATC
NLR family pyrin domain containing 3	*NLRP3* (NM_001079821.3)	Forward: TGGCATCGTGAAGTGGTTGT<br>Reverse: AGCCAAATGCTTACCAGAAAGT
PYD and CARD domain containing	*ASC* (NM_013258.5)	Forward: TGGATGCTCTGTACGGGAAG<br>Reverse: CCAGGCTGGTGTGAAACTGAA
Caspase 1	*CASP1* (NM_001223.5)	Forward: TCCAATAATGGACAAGTCAAGCC<br>Reverse: GCTGTACCCCAGATTTTGTAGCA
Interleukin 1 beta	*IL1β* (NM_000576.3)	Forward: AGCTACGAATCTCCGACCAC<br>Reverse: CGTTATCCCATGTGTCGAAGAA
Interleukin 18	*IL18* (NM_001243211.2)	Forward: TCTTCATTGACCAAGGAAATCGG<br>Reverse: TCCGGGGTGCATTATCTCTAC
Interleukin 6	*IL6* (NM_001371096.1)	Forward: CCTGAACCTTCCAAAGATGGC<br>Reverse: TTCACCAGGCAAGTCTCCTCA
Tumor necrosis factor	*TNFα* (NM_000594.4)	Forward: CCTCTCTCTAATCAGCCCTCTG<br>Reverse: GAGGACCTGGGAGTAGATGAG
Toll like receptor 2	*TLR2* (NM_001318787.2)	Forward: ATCCTCCAATCAGGCTTCTCT<br>Reverse: GGACAGGTCAAGGCTTTTTACA
Toll like receptor 4	*TLR4* (NM_003266.4)	Forward: CTTGGCCCTAAACCACACAGA<br>Reverse: CAATGGAATCGGGGTGAAGG
Matrix metallopeptidase 2	*MMP2* (NM_001302510.2)	Forward: GATACCCCTTTGACGGTAAGGA<br>Reverse: CCTTCTCCCAAGGTCCATAGC

Matrix metallopeptidase 9	*MMP9* (NM_004994.3)	Forward: CGCAGACATCGTCATCCAGT<br>Reverse: GAAATGGGCGTCTCCCTGAA
Tissue inhibitor of metalloproteinase 1	*TIMP1* (NM_003254.3)	Forward: AAT TCC GAC CTC GTC ATC AG<br>Reverse: GTT TGC AGG GGA TGG ATA AA
Tissue inhibitor of metalloproteinase 2	*TIMP2* (NM_003255.5)	Forward: CTG GAC GTT GGA GGA AAG AA<br>Reverse: GTC GAG AAA CTC CTG CTT GG
